# Matching Imaging and Remodulation Effects: Benefits of Cardiac Contractility Modulation Shown by Global Longitudinal Strain: A Case Report

**DOI:** 10.3390/clinpract12010015

**Published:** 2022-02-17

**Authors:** Andrea Matteucci, Giacomo Bonacchi, Vincenzo M. La Fazia, Giuseppe Stifano, Domenico Sergi

**Affiliations:** 1 Division of Cardiology, San Filippo Neri Hospital, Via Martinotti, 20, 00135 Rome, Italy; 2 Division of Cardiology, University Hospital “Tor Vergata”, 00133 Rome, Italy; giacomobonacchi1@gmail.com (G.B.); vmirco.lafazia@gmail.com (V.M.L.F.); giuseppe.stifano@hotmail.it (G.S.); domenicosergi@gmail.com (D.S.)

**Keywords:** GLS, cardiac contractility modulation, genetic rearrangement, QOL

## Abstract

Cardiac Contractility Modulation (CCM) has been proposed for inpatients affected by heart failure with reduced ejection fraction (HFrEF), with relapsing HF symptoms. We present a case of a patient treated with percutaneous coronary intervention (PCI) in the setting of acute coronary syndrome without persistent ST-segment elevation, with the best medical therapy for decompensated HF. The patient refused the implantable cardioverter-defibrillator (ICD), and to reduce the increasing number of hospitalizations for HF exacerbations, we proposed the use of the cardiac contractility modulation device. After the implant, the patient demonstrated a marked improvement in exercise effort and quality of life (QOL) with a six-minute walk test (SMWT), Minnesota Living with Heart Failure Questionnaire (MLWHFQ), and echocardiographic parameters. At 9 months after discharge, no hospital admissions for HF were recorded. We showed with the speckle tracking imaging how the improvement in global longitudinal strain (GLS) correlates with the remodeling effects on myocardial cells.

## 1. Introduction

Cardiac Contractility Modulation (CCM) is an innovative therapeutic opportunity for patients affected by heart failure with reduced ejection fraction (HFrEF) [1]. It affects the epigenetic and proteomic landscape of cardiomyocytes, providing an increase in patients’ exercise capacity and quality of life and reducing hospital admissions. Analysis of myocardial tissue from both animal models and human hearts treated by CCM demonstrates a shift of abnormally expressed genes towards normal function, positively affecting pathways involving proteins that regulate calcium cycling and myocardial contraction. CCM effects are proven to be independent of QRS duration; however, clinical studies to date have primarily focused on patients with normal QRS since cardiac resynchronization therapy is a well-established option for patients with heart failure and a prolonged QRS duration [2]. We show the usefulness of speckle tracking strain imaging [3] in confirming the clinical improvement in these patients.

## 2. Case Report

**Figure 1:**
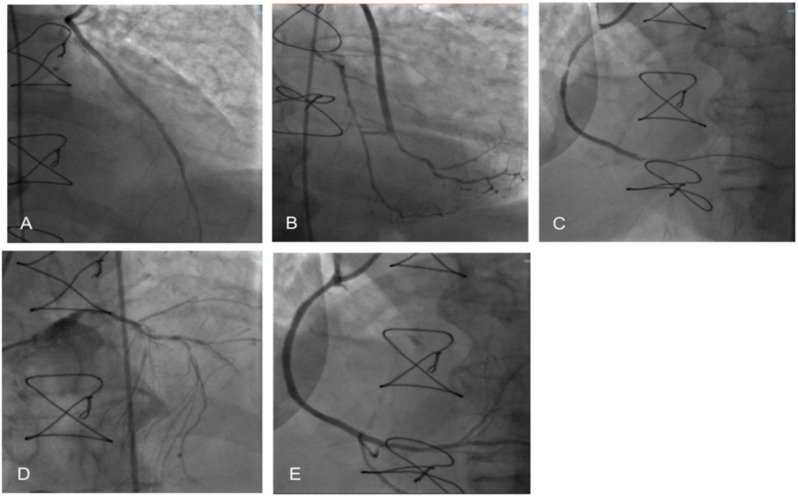
Percutaneous coronary intervention. (**A**) Internal mammary artery on anterior descending artery; (**B**) Y venous graft on an obtuse marginal branch and posterior descending artery; (**C**) Right coronary artery proximal and distal occlusion; (**D**) Left main coronary artery; (**E**) Right coronary artery after percutaneous angioplasty and drug-eluting stent implantation.

We present the case of a 77-year-old patient, admitted to our Cardiology Department for worsening rest dyspnea. The patient had a medical history of chronic coronary syndrome, triple coronary artery bypass grafts, hypertension chronic obstructive pulmonary disease, insulin-treated type 2 diabetes mellitus, and dyslipidemia. Over the past 12 months, she was admitted to the hospital thrice for HF recurrences. On these occasions, a severely depressed systolic function with 30% left ventricular ejection fraction (LVEF) was documented, associated with exertional dyspnea and asthenia. In her medical history, it was also reported she was proposed to undergo ICD implantation, but she refused due to fear and anxiety of potential shocks delivered by the device. It should be noted, however, that despite the ischemic etiology of cardiomyopathy, neither sustained arrhythmias nor frequent ventricular ectopic beats were reported in her medical history. At the current hospital admission, she presented arterial blood pressure values of 100/65 mmHg, leg edema, and elevated jugular venous pressure from the observation of the right side of the patient’s neck. The medical therapy on admission was appropriately optimized according to the patient’s condition (acetylsalicylic acid, angiotensin receptor blocker, beta-blocker, statin, diuretic, and mineralocorticoid receptor antagonist). The first echocardiographic evaluation showed akinesia of the apex and all distal segments, hypokinesia of the anterior wall and interventricular septum, with severely reduced global systolic function, LVEF 25%, and mild mitral regurgitation. The patient underwent coronary angiography, which showed the patent left internal mammary artery bypass graft and saphenous vein bypass graft (Figure 1); a new non-documented distal right coronary artery occlusion was found, and then treated with percutaneous angioplasty and the implantation of one drug-eluting stent. 

**Figure 2:**
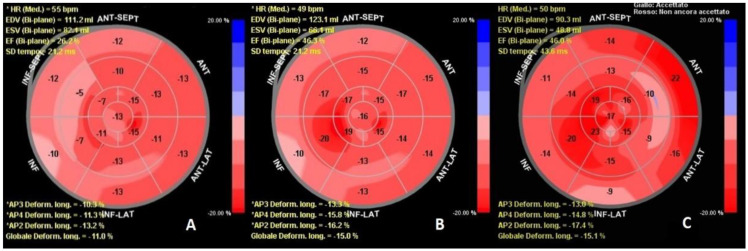
Left ventricular global longitudinal strain progression before and after implantation. (**A**) Left ventricular global longitudinal strain acquired the day before CCM implantation. (**B**) 30 days’ follow-up; the mid-segments of the infero-septal regions showed the most improvement from the device-induced remodeling. (**C**) 6 months follow-up; apex, basal anterior septum, and basal anterolateral wall have further improved. As can be seen in the image, end-diastolic volume (EDV) has not been changed consistently for one month. It is reduced compared with the implantation after 6 months. The end-systolic volume (ESV) reduced immediately after the procedure. This may be due to increased inotropism and lusipropism as a result of cardiac contractility modulation therapy.

She was treated with a loop diuretic and dopamine infusion, gradually tapered during her hospital stay. On day 10, a new acute pulmonary edema event on transitory high arterial pressure occurred. On day 40, we evaluated the patient LVEF with echocardiographic speckle tracking strain imaging, a quantitative technique to estimate myocardial function through a non-Doppler angle-independent objective analysis of myocardial deformation [4]. The analysis demonstrated AP3 longitudinal deformation (LD) −10.3%, AP2 LD −11.3%, AP4 LD −13.2%, global LD −11.0%. The LVEF of 26% calculated with biplane Simpson’s formula (Figure 2A), did not show any improvement from the time of the hospitalization onset. We evaluated the patient’s exercise effort and quality of life (QOL) with a six-minute walk test (SMWT), and Minnesota Living with Heart Failure Questionnaire (MLWHFQ). The results consisted of a distance of 50 m walked, with desaturation of 88% and a questionnaire score of 84 points. In consideration of the persistence of severely reduced LVEF, the recurrent hospital admissions for HF, and the ineligibility for cardiac resynchronization therapy due to QRS < 130 ms, we proposed the implantation of the CCM device. It was accepted as the patient was reassured that, differently from ICD, CCM device cannot deliver high energy shocks and is focused on HF symptoms treatment through the delivery of daily electric therapy that cannot be perceived. Furthermore, as previously reported, the patient did not require pacing or resynchronization therapy due to the normal QRS duration. The discharge medications of the patient were acetylsalicylic acid 100 mg, telmisartan 80 mg, bisoprolol 1.25 mg (reduced from 3.75 mg to 1.25 mg during hospitalization due to bradycardia), atorvastatin 80 mg, furosemide 125 mg, and canrenone 50 mg. Angiotensin receptor/neprilysin inhibitor was not added to medical therapy due to frequent episodes of hypotension; furthermore, sodium-glucose cotransporter-2 inhibitors were not recommended due to persistent impaired renal function (eGFR of 44 mL/min/1.73 m^2^) and a previous diabetic ketoacidosis episode.

One week after the discharge, optimization of device parameters was performed, with an increase in daily therapy stimulation hours provided by the device up to 12 h per day. At 1-month follow-up, she referred a marked improvement in symptoms and exercise dyspnea. The SMWT distance increased to 150 m, without desaturation, and the MLWHFQ score improved to 63. The echocardiographic evaluation of LV strain showed an apical three-chamber view value of −13.3%, a two-chamber view value of −15.8%, and a four-chamber view value of −16.2% with a global result of −15.0%, with the highest increase in the stimulated regions (Figure 2B).

The 3-months follow-up evaluation showed additional improvement: MLWHFQ score reduced to 58 points and SMWT distance reached 230 m without desaturation. We reduced diuretic dosage from furosemide 125 mg b.i.d. to 25 mg t.i.d. At 6 months we confirmed improvement in LV strain parameters, with 46% LVEF calculated with GLS (Figure 2C). Finally, the QOL test results also confirmed the improvements observed in the third month of follow-up. The SMWT distance was 210 m without desaturation, and the MLWHFQ score was 52. At 9 months after discharge, no hospital admissions for HF were recorded.

## 3. Discussion

In the current case, the CCM implantation aimed to improve symptoms and to reduce hospitalization in a patient affected by HFrEF. It offers also the possibility to reconsider ICD implantation indications after patient refusal during the hospital stay. In addition, the patient had a normal QRS duration, and she did not require pacing. This, by the patient’s will, allowed us to choose the most suitable device for the patient’s needs, avoiding secondary effects from the ICD and resynchronization therapy. In fact, in addition to the possibility of inappropriate shocks from the ICD, resynchronization therapy can lead to scar formation and myocardial cells apoptosis in the context of dyssynchrony [5]. This case shows the efficacy of GLS analysis in revealing the improvement of LV performance concurrent with clinical evaluation. The CCM system provides high voltage biphasic electrical impulses during the absolute ventricular refractory period. It uses common electrophysiological tolls and two leads approach on the interventricular septum. Rather than eliciting new contractions, these signals influence the biology of the failing myocardium, increasing contractile strength without enhancing myocardial oxygen consumption. The signals have been shown to normalize the phosphorylation of regulatory proteins such as Phospholamban (PLB) in vitro, within seconds of treatment. Improvement in calcium handling via sarco-endoplasmic reticulum calcium ATPase (SERCA2a) upregulation and restoration of the sodium/calcium exchanger further increases contractile function in subjects with heart failure. This improves calcium handling and increases ventricular contractility with resultant improvement in exercise tolerance and functional capacity [6]. In our case, we observed only a small improvement in LVEF and clinical condition after 40 days from the revascularization of a chronically closed vessel (right coronary artery). The segments with improved contractility were the anterior septum, anterior wall, and apex, as the acute molecular effects of CCM mostly affected the local area of signal delivery [7]. Several hypotheses can be advanced on the contribution of PCI on LVEF improvement. We cannot exclude the contribution of RCA revascularization; however, in contrast to this assumption, the patient previously presented two independent echocardiographic reports documenting a 33% and 30% LVEF in June and November 2019, respectively. According to current guidelines, this patient was indicated for the implantation of an ICD for arrhythmic risk protection. As it was not possible to perform the implantation due to the patient’s will, the adoption of the CCM device allowed at least to alleviate the HF symptoms, resulting in an improved LVEF which outperformed the indications for ICD implantation. In addition, the myocardial wall segments showing the greater remodeling therapy effects were areas whose circulation was supplied by the left coronary artery. The contribution of the revascularization of an existing diseased native vessel remains controversial, but we cannot exclude that it played a role in the recovery. There is also a lack of evidence in the literature of the existence of so-called “super responders”. Indeed, as for CRT patients, we cannot exclude that several patients may have a greater benefit compared with the one reported in the limited literature, with an increase in ejection fraction higher than the regular 10% reported by the literature. More studies are needed to confirm the benefit of CCM therapy in cardiac reverse remodeling. To the best of our knowledge, this is the first case that evaluated the effects of CCM remodeling using GLS. We suggest the prevalent role of CCM on top of optimal medical therapy in the echocardiographic and clinical improvement during the follow-up. In this case, stimulated regions showed the best results, correlating with the areas undergoing the most significant genetic rearrangement. Furthermore, the de-escalation of diuretic therapy during the follow-up demonstrated the improvement of myocardial contraction in the setting of HFrEF. Our patient returned to NYHA class II, being able to carry out many activities of daily living which she had ceased, improving her QOL. Left ventricular strain analysis proved to be useful and effective in monitoring the progress of CCM therapy by matching imaging and myocardial gene rearrangements induced by the device.

## Data Availability

Data supporting reported results can be found by contacting the Department of Cardiology of Policlinico Tor Vergata, Rome, Italy, Tel.: +39-06-2090-4044.
